# Effects of oral oligopeptide preparation and exercise intervention in older people with sarcopenia: a randomized controlled trial

**DOI:** 10.1186/s12877-024-04860-2

**Published:** 2024-03-18

**Authors:** Xinyi Liao, Daomei Cheng, Jingjing Li, Lin Zhu, Suqiong Zhang, Xiaofan Jing, Lei Shi

**Affiliations:** 1 Department of Clinical Nutrition, West China Hospital, Sichuan University, Sichuan Province, Chengdu, China; 2 National Clinical Research Center for Geriatrics, West China Hospital, Sichuan University, Chengdu, Sichuan Province China; 3 School of Public Health, Chengdu Medical College, Sichuan Province, Chengdu, China; 4 Zhengxing Community Health Service Center of Tianfu New District, Sichuan Province, Chengdu, China; 5 Shibantan Community Health Service Center of Xindu District, Sichuan Province, Chengdu, China

**Keywords:** Older people, Sarcopenia, Peptide, Nutrition

## Abstract

**Background:**

Nutrition and exercise are important interventions for sarcopenia. There were few studies on oral oligopeptide nutrition preparations combined with exercise to intervene in the older people with sarcopenia. The aim of this study was to verify the effectiveness of oligopeptide nutrition preparation combined with exercise intervention on the older people with sarcopenia in community.

**Methods:**

A total of 219 subjects aged 65 years or older with sarcopenia were randomly divided into 4 groups. The nutrition group (*n* = 58) was given individualized nutrition education and oral oligopeptide nutrition preparation. The exercise group (*n* = 50) received exercise intervention. The combined group (*n* = 52) received both oral nutrition preparation and exercise interventions. The control group (*n* = 59) only received individualized nutrition education. The nutrition preparation can provide energy 185kcal and protein 24.2g per day. The exercise intervention including warm-up exercise, resistance exercise and aerobic exercise, the training time was 60min for 5 times every week. The intervention lasted for 16 weeks. Hand grip strength, gait speed, body composition and hematology parameters were measured before and after intervention.

**Results:**

A total of 159 subjects completed the study. Compared with baseline, the left grip strength and 6-m walking speed of the subjects in nutrition group increased significantly after the intervention, and the grip strength of both hands in exercise group and combined group increased significantly. The body weight of the subjects in nutrition group, exercise group and combined group increased significantly after intervention, but no increase in soft lean mass (SLM) and skeletal muscle mass (SMM) was observed in any of the four groups. The fat-free mass (FFM) of the legs of the control group, exercise group and nutrition group decreased after intervention, and only the FFM of the legs of the combined group maintained the level before the intervention.

**Conclusion:**

Both oral peptide nutrition and exercise interventions can improve the muscle strength or function of the older people with sarcopenia. However, there were no increases in muscle mass observed.

**Trial registration:**

ChiCTR, ChiCTR2100052135. Registered 20 October 2021, https://www.chictr.org.cn/showproj.html?proj=135743

**Supplementary Information:**

The online version contains supplementary material available at 10.1186/s12877-024-04860-2.

## Background

Sarcopenia was defined as age-related loss of muscle mass, plus low muscle strength, and/or low physical performance [1]. Data have shown that the prevalence of sarcopenia in the older population over 65 years in Asia is about 5.5%—25.7% [2]. The prevalence of sarcopenia in older adults in China was approximately 18% in men and 16.4% in women [3]. It is expected that by 2050, the number of people over 65 years old in China will increase to 366 million. The severe aging trend has increased the attention given to age-related diseases such as sarcopenia.

Sarcopenia may increase the incidence of falls and fractures in the older population, increase the risk of disability [4], seriously affect the quality of life and health status, and impose a heavy burden on the health care system and society [5]. Exercise and nutrition are important interventions for sarcopenia [2, 6]. Available evidence suggests that interventions which included resistance exercise plus nutritional supplements can significantly increase physical function, muscle mass and strength [2].

Regular activity especially resistance exercise is essential for healthy aging and offers a range of health benefits [7], but older people have difficulty achieving or not achieve 150 min of physical activity per week recommended activity level to maintain or improve health-related levels of physical fitness. Therefore, it is crucial to find safe and effective exercise methods and carry out sustainable exercise intervention for older population [8].

In studies on nutritional intervention for older people with sarcopenia, the most commonly used protein sources include whey protein, casein and soy protein. Compared with proteins, oligopeptides have faster transport speed and lower energy consumption, can eliminate absorption competition with free amino acids, and have higher digestibility and utilization.

In this study, the older people with sarcopenia recruited from rural areas in Chengdu, Sichuan Province, were randomly divided into 4 groups. Exercise and nutrition intervention were given to observe the changes in muscle strength, muscle quality and activity ability. The intervention preparation used in the study was a compound enteral nutrition preparation with oligopeptide as the main protein source. The purpose of this study was to verify the effectiveness of compound oligopeptide enteral nutrition preparation alone or in combination with exercise in the older population with sarcopenia.

## Method

### Study design

**Fig.1:**
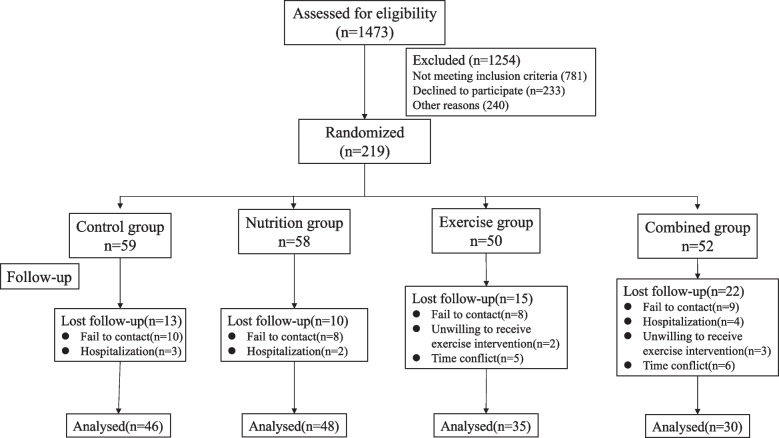
Flow chart of the study

This is a multicenter randomized controlled study. A total of 219 older people with sarcopenia were randomly divided into 4 groups: the control group was given individualized nutrition education provided by professional dietitians; the nutrition group was given nutrition education and oral enteral nutrition preparations rich in oligopeptides; the exercise group received exercise intervention and nutrition education; and the combined group received both nutrition and exercise intervention. The intervention lasted for 16 weeks. A 24-h dietary survey was conducted at the baseline and end of the intervention. Muscle strength, physical function and body composition were measured before and after the intervention, and fasting blood samples were collected for hematological examination, such as routine blood tests, fasting blood glucose, blood lipids and liver and kidney function. The study was carried out in the health centers of the community. A total of 219 subjects were included in the project, and the flow chart of the experiment is shown in Fig. 1. Due to the exercise intervention used in this study, the subjects and researchers were not blinded.

This study was approved by the Ethics Committee on Biomedical Research, West China Hospital of Sichuan University and registered at https://www.chictr.org.cn/ (ChiCTR2100052135). All participants provided written informed consent.

### Outcomes

The primary outcome was the change in grip strength and 6-m gait speed over 16 weeks. Secondary outcomes included the change of dietary intake, body composition and hematological indexes between the baseline and after intervention.

### Nutrition intervention and supplement protocol

At the beginning of the study, all subjects received nutrition education provided by professional dietitians. Subjects in the nutrition group and combined group received nutrition intervention of oral oligopeptide preparation twice a day for 25 g each time. Subjects in the nutrition group intake the preparation after breakfast and lunch, and those in the combined group intake the preparation once after exercise within half an hour and once after lunch.

The enteral nutrition preparation used in this study was an enteral nutrition preparation with plant oligopeptides as the main protein sources developed in the previous study. It can provide energy 185 kcal, protein 24.2 g (including plant oligopeptide 11 g, casein peptide 4 g, branch chain amino acid 5 g), CaHMB2.5 g per day.

### Exercise intervention

Subjects in the exercise group and combined group received the exercise intervention. The exercise program used in this study was designed by professional rehabilitation doctors considering the physical activity ability of older people with sarcopenia, including warm-up exercise, resistance exercise and relaxation. The exercise program needs to be completed by dumbbells. The exercise intervention was organized by community doctors and assisted by staff with experience in sports rehabilitation. Exercise was performed 5 times a week in the activity room of the community health service center, each lasting 45 to 60 min.

### Sample size calculation

The sample size estimation was based on anticipated differences in gait speed of the subjects [9], with alpha = 0.05 and power = 0.8 (two-sided). The estimated minimum sample size was 26 subjects per group. However, to avoid the influence of dropout rate, we opted to include more participants.

### Statistical analysis

Epidata 3.1 was used for data entry, and SPSS 27.0 was used for statistical analysis of the data. The classification data were described by rate (%), the quantitative data were described by the mean (standard deviation) [$\overline{x }$(s)]. The Pearson chi-square test, Fisher’s exact test and one-way ANOVA were used to compare the differences between the groups at baseline. The Paired Samples *t*-test was used for comparison before and after intervention. All statistical tests were two-tailed, and significance was set at *p* < 0.05.

## Results

### Baseline and demographic data

A total of 159 subjects completed the study and were included in the statistical analysis. There was no significant difference in the average age, sex, disease history or MNA-SF score among groups, but the right hand grip strength of the nutrition group was significantly higher than that of the control group and exercise group (Table 1).

**Table 1:** Demographic characteristics and baseline data of subjects

	Control group(*n* = 46)	Exercise group(*n* = 35)	Nutrition group(*n* = 48)	Combined group(*n* = 30)	*P* value
**Age(years)[** $\overline{{\varvec{x}} }$**(s)]**	73.21(4.98)	72.04(5.02)	72.68(5.59)	70.52(3.30)	0.209
**Gender(female%)**	22(47.8%)	19(54.3%)	23(47.9%)	14(46.7%)	0.918
**Baseline data[** $\overline{{\varvec{x}} }$**(s)]**					
Body weight(kg)	49.54 (7.02)	46.59 (6.70)	49.18 (7.36)	51.52 (6.43)	0.105
BMI (kg/m^2^)	21.86(2.57)	21.47(3.45)	21.49(2.80)	22.53(2.28)	0.526
Left grip strength(kg)	21.02(6.84)	19.52(3.75)	21.92(6.41)	21.91(6.21)	0.391
Right grip strength(kg)	20.50(5.27)	20.11(3.73)	23.78(6.85)^*^^#^	23.06(7.05)	0.044
6-m walking speed(m/s)	0.92(0.18)	0.99(0.17)	0.96(0.23)	1.05(0.18)	0.139
SMM (kg)	17.40(3.29)	16.51(2.19)	17.93(3.58)	18.05(3.10)	0.260
SMI (kg)	5.54(0.75)	5.40(0.52)	5.56(0.82)	5.71(0.77)	0.524
MNA-SF score	12.75(1.16)	12.85(0.99)	12.59(1.12)	13.08(0.95)	0.666
**Disease history[n(%)]**					
Hypertension	14(30.43%)	11(31.43%)	14(29.17%)	10(33.33%)	0.984
Diabetes mellitus	9(19.57%)	9(25.71%)	12(25.00%)	5(16.67%)	0.756
Lipid abnormality	8(17.39%)	8(22.86%)	10(20.83%)	4(13.33%)	0.765
Osteoporosis	1(2.17%)	1(2.86%)	1(2.08%)	2(6.67%)	0.729
Fracture history	3(6.52%)	4(11.43%)	3(6.25%)	2(6.67%)	0.858

^*^The difference was statistically significant (*P* < 0.05) compared with the control group

^#^The difference was statistically significant (*P* < 0.05) compared with the exercise group

### Dietary intake of subjects before and after intervention

Compared with the baseline, the daily dietary energy and protein intake of the four groups showed an upwards trend after intervention, and the daily dietary energy intake of the control group and the nutrition group was significantly higher than that before the intervention (*P* < 0.05) (Table 2).

**Table 2:** Dietary and total intake before and after the intervention

	Baseline	Week 16			
	Diet intake	Diet intake	*P* value ^a^	Total intake	*P* value ^b^
**Control group**					
Energy, kcal	1084.80 ± 380.60	1357.94 ± 549.91	0.026	/	/
Energy, kcal/(kg·d)	21.42 ± 7.23	25.99 ± 8.52	0.032	/	/
Protein, g	34.88 ± 23.65	39.95 ± 23.96	0.235	/	/
Protein, g/(kg·d)	0.70 ± 0.51	0.76 ± 0.46	0.425	/	/
**Exercise group**					
Energy, kcal	1320.73 ± 441.23	1499.09 ± 509.30	0.115	/	/
Energy, kcal/(kg·d)	28.40 ± 9.64	31.30 ± 10.11	0.227	/	/
Protein, g	38.12 ± 14.16	48.79 ± 26.98	0.092	/	/
Protein, g/(kg·d)	0.82 ± 0.36	1.01 ± 0.54	0.165	/	/
**Nutrition group**					
Energy, kcal	1195.42 ± 507.48	1446.91 ± 505.90	0.006	1631.91 ± 505.90	< 0.001
Energy, kcal/(kg·d)	24.39 ± 10.14	28.60 ± 8.91	0.022	32.35 ± 8.87	< 0.001
Protein, g	39.11 ± 24.20	43.77 ± 27.22	0.210	67.77 ± 27.22	< 0.001
Protein, g/(kg·d)	0.79 ± 0.44	0.86 ± 0.43	0.333	1.34 ± 0.42	< 0.001
**Combined group**					
Energy, kcal	1286.05 ± 593.56	1432.00 ± 535.94	0.274	1617.00 ± 535.94	0.019
Energy, kcal/(kg·d)	24.84 ± 10.69	26.60 ± 8.00	0.475	30.16 ± 7.81	0.038
Protein, g	41.12 ± 19.83	45.41 ± 23.60	0.449	69.41 ± 23.60	< 0.001
Protein, g/(kg·d)	0.80 ± 0.39	0.83 ± 0.35	0.756	1.29 ± 0.32	< 0.001

Values were expressed as mean ± SD; the total intake after intervention was the sum of dietary intake and oral peptide preparations intake at 16 weeks of intervention

^a^represents the within-group change of diet intake from the baseline to 16 weeks of intervention

^b^represents the within-group change between diet intake at the baseline and the total intake at 16 weeks of intervention

### Changes in muscle strength and physical function before and after intervention

After the intervention, the left hand grip strength and walking speed of the nutrition group were significantly increased. The grip strength of the exercise group and the combined group was significantly increased, but no significant increase in walking speed was observed in these 2 groups (Table 3).

**Table 3:** Grip strength, walking speed and body composition before and after intervention

	Baseline	Week 16	*t* value	*P* value
**Body weight (kg)**				
Control group	49.54 ± 7.02	50.01 ± 7.44	-0.998	0.328
Exercise group	46.59 ± 6.70	47.33 ± 6.51	-2.721	0.011
Nutrition group	49.18 ± 7.36	50.25 ± 7.33	-3.955	0.000
Combined group	51.52 ± 6.43	52.74 ± 7.11	-3.960	0.001
**Body mass index (BMI) (kg/m**^**2**^**)**				
Control group	21.86 ± 2.57	22.05 ± 2.44	-0.957	0.348
Exercise group	21.47 ± 3.45	21.82 ± 3.30	-2.865	0.008
Nutrition group	21.49 ± 2.80	21.95 ± 2.75	-3.898	0.000
Combined group	22.53 ± 2.28	23.05 ± 2.45	-3.910	0.001
**Left grip strength (kg)**				
Control group	21.02 ± 6.84	21.30 ± 5.97	-0.574	0.571
Exercise group	19.52 ± 3.75	20.99 ± 3.87	-2.512	0.019
Nutrition group	21.92 ± 6.41	23.49 ± 6.90	-2.962	0.005
Combined group	21.91 ± 6.21	24.65 ± 6.56	-3.385	0.003
**Right grip strength (kg)**				
Control group	20.50 ± 5.27	21.12 ± 4.56	-1.537	0.137
Exercise group	20.11 ± 3.73	22.21 ± 4.56	-2.789	0.010
Nutrition group	23.78 ± 6.85	24.58 ± 6.52	-1.355	0.184
Combined group	23.06 ± 7.05	25.11 ± 6.56	-2.168	0.042
**6-m walking speed (m/s)**				
Control group	0.92 ± 0.18	0.96 ± 0.16	-0.756	0.457
Exercise group	0.99 ± 0.17	1.06 ± 0.20	-1.379	0.180
Nutrition group	0.96 ± 0.23	1.10 ± 0.21	-3.908	0.000
Combined group	1.05 ± 0.18	1.02 ± 0.24	0.536	0.598
**Skeletal muscle mass (SMM)(kg)**				
Control group	17.40 ± 3.29	17.41 ± 3.20	-0.061	0.952
Exercise group	16.51 ± 2.19	16.39 ± 2.00	0.697	0.492
Nutrition group	17.93 ± 3.58	18.05 ± 3.65	-0.966	0.341
Combined group	18.05 ± 3.10	18.45 ± 3.25	-1.800	0.086
**Appendicular skeletal muscle mass index (SMI)**				
Control group	5.54 ± 0.75	5.43 ± 0.79	2.042	0.052
Exercise group	5.40 ± 0.52	5.25 ± 0.49	3.022	0.006
Nutrition group	5.56 ± 0.82	5.53 ± 0.79	1.090	0.283
Combined group	5.71 ± 0.77	5.73 ± 0.80	-0.367	0.717
**Appendicular skeletal muscle mass (ASMM)(kg)**				
Control group	12.70 ± 2.86	12.47 ± 2.88	1.920	0.067
Exercise group	11.77 ± 1.63	11.47 ± 1.59	2.885	0.008
Nutrition group	12.92 ± 3.08	12.82 ± 3.02	1.264	0.214
Combined group	13.18 ± 2.66	13.21 ± 2.76	-0.293	0.772
**Fat free mass of left arm(kg)**				
Control group	1.52 ± 0.36	1.53 ± 0.34	-0.343	0.734
Exercise group	1.44 ± 0.27	1.44 ± 0.24	-0.041	0.968
Nutrition group	1.61 ± 0.42	1.63 ± 0.43	-1.049	0.301
Combined group	1.60 ± 0.37	1.66 ± 0.37	-1.690	0.106
**Fat free mass of right arm(kg)**				
Control group	1.55 ± 0.39	1.55 ± 0.35	-0.075	0.941
Exercise group	1.45 ± 0.30	1.45 ± 0.26	0.076	0.940
Nutrition group	1.65 ± 0.43	1.66 ± 0.43	-0.576	0.568
Combined group	1.67 ± 0.41	1.70 ± 0.39	-0.795	0.436
**Fat free mass of trunk(kg)**				
Control group	14.89 ± 2.51	15.00 ± 2.32	-0.712	0.483
Exercise group	14.20 ± 1.83	14.29 ± 1.66	-0.588	0.562
Nutrition group	15.42 ± 2.82	15.58 ± 2.80	-1.595	0.119
Combined group	15.55 ± 2.49	15.85 ± 2.49	-1.713	0.101
**Fat free mass of left leg(kg)**				
Control group	4.79 ± 1.09	4.68 ± 1.12	3.031	0.006
Exercise group	4.43 ± 0.59	4.28 ± 0.60	4.117	0.000
Nutrition group	4.81 ± 1.14	4.75 ± 1.11	2.613	0.013
Combined group	4.92 ± 0.98	4.89 ± 1.01	1.002	0.328
**Fat free mass of right leg(kg)**				
Control group	4.83 ± 1.09	4.70 ± 1.12	3.165	0.004
Exercise group	4.44 ± 0.57	4.30 ± 0.58	4.011	0.000
Nutrition group	4.86 ± 1.15	4.79 ± 1.13	2.302	0.027
Combined group	4.98 ± 0.99	4.96 ± 1.04	0.500	0.622
**Soft lean mass (SLM) (kg)**				
Control group	31.34 ± 5.21	31.23 ± 5.16	0.340	0.737
Exercise group	29.89 ± 3.43	29.56 ± 3.11	1.170	0.253
Nutrition group	32.11 ± 5.68	32.24 ± 5.77	-0.639	0.527
Combined group	32.27 ± 4.94	32.80 ± 5.13	-1.572	0.131
**Body cell mass (BCM)**				
Control group	21.30 ± 3.61	21.32 ± 3.52	-0.092	0.927
Exercise group	20.34 ± 2.40	20.21 ± 2.20	0.653	0.520
Nutrition group	21.88 ± 3.92	22.02 ± 3.98	-1.015	0.317
Combined group	22.01 ± 3.41	22.46 ± 3.57	-1.950	0.065

### Changes in body weight and body composition before and after intervention

After the intervention, the body weight and BMI of the exercise group, nutrition group and combined group increased significantly (*P* < 0.05). The weight and BMI of the control group increased, but there was no significant difference before and after the intervention.

There was no significant increase in soft lean mass (SLM) or skeletal muscle mass (SMM) before and after intervention in the four groups. The appendicular skeletal muscle mass (ASMM) and appendicular skeletal muscle mass index (SMI) decreased after intervention in the exercise group, but there was no significant difference in the other three groups before and after intervention. The fat free mass (FFM) of the left arm, right arm and trunk of the four groups did not change significantly before and after intervention, but the FFM of the left leg and right leg of the control group, exercise group and nutrition group decreased significantly after intervention, while the FFM of both legs of the combined group maintained the level before intervention. There was no significant change in BCM in the four groups before and after intervention.

### Changes in hematology before and after intervention

The hematological indexes before and after intervention are shown in Table 4. There were no significant changes in red blood cells and hemoglobin before and after intervention in the four groups. After 16 weeks of intervention, the levels of serum albumin in nutrition group (*P* = 0.022) and combined group (*P* = 0.039) increased significantly, but there was no significant change in control group and exercise group. After the intervention, the fasting blood glucose of the nutrition group increased significantly (*p* = 0.037), but the value was within the normal range before and after the intervention. There was no significant change in the fasting blood glucose of the other groups before and after the intervention. There was no significant change in blood lipid levels before and after the intervention.

**Table 4:** Comparison of hematological parameters before and after intervention

	**Baseline**	**Week 16**	***p *value**
**Red blood cell (10**^**12**^**/L)**			
Control group	4.11 ± 0.38	4.14 ± 0.31	0.581
Exercise group	4.05 ± 0.16	4.29 ± 0.12	0.254
Nutrition group	4.42 ± 0.42	4.45 ± 0.38	0.756
Combined group	4.45 ± 0.33	4.60 ± 0.45	0.464
**Hemoglobin (g/L)**			
Control group	128.27 ± 9.15	130.13 ± 8.31	0.257
Exercise group	125.33 ± 6.66	132.33 ± 6.17	0.149
Nutrition group	137.50 ± 12.83	140.20 ± 11.61	0.167
Combined group	129.60 ± 10.78	134.40 ± 9.63	0.244
**Albumin(g/L)**			
Control group	45.22 ± 1.27	44.57 ± 1.48	0.243
Exercise group	44.00 ± 5.77	44.17 ± 1.29	0.961
Nutrition group	43.00 ± 2.85	45.15 ± 1.85	0.022
Combined group	43.21 ± 2.59	45.93 ± 2.28	0.039
**Fasting blood glucose (mmol/L)**			
Control group	5.74 ± 0.65	5.78 ± 0.68	0.858
Exercise group	5.93 ± 0.19	6.29 ± 0.39	0.174
Nutrition group	5.18 ± 0.43	5.49 ± 0.34	0.037
Combined group	5.77 ± 0.58	6.43 ± 1.43	0.293
**Triglyceride (mmol/L)**			
Control group	1.28 ± 0.63	1.43 ± 0.75	0.289
Exercise group	1.24 ± 0.44	1.50 ± 0.29	0.132
Nutrition group	1.12 ± 0.71	1.05 ± 0.75	0.806
Combined group	0.97 ± 0.31	1.29 ± 0.47	0.176
**Total cholesterol (mmol/L)**			
Control group	5.08 ± 1.15	5.26 ± 1.09	0.410
Exercise group	4.93 ± 0.68	5.34 ± 0.45	0.331
Nutrition group	4.97 ± 1.01	4.96 ± 0.69	0.954
Combined group	4.46 ± 0.79	4.65 ± 0.91	0.487
**High density lipoprotein cholesterol (mmol/L)**			
Control group	1.92 ± 0.42	1.85 ± 0.43	0.358
Exercise group	1.76 ± 0.14	1.74 ± 0.07	0.705
Nutrition group	1.86 ± 0.66	1.83 ± 0.46	0.749
Combined group	1.53 ± 0.41	1.48 ± 0.34	0.480
**Low density lipoprotein cholesterol (mmol/L)**			
Control group	2.59 ± 0.97	2.75 ± 0.99	0.329
Exercise group	2.61 ± 0.38	2.92 ± 0.38	0.384
Nutrition group	2.60 ± 0.69	2.65 ± 0.63	0.824
Combined group	2.48 ± 0.47	2.58 ± 0.55	0.555
**Alanine aminotransferase (U/L)**			
Control group	20.40 ± 9.77	19.73 ± 8.99	0.703
Exercise group	19.67 ± 9.07	19.67 ± 4.04	1.000
Nutrition group	23.40 ± 10.78	33.20 ± 25.09	0.199
Combined group	23.80 ± 24.78	23.00 ± 10.58	0.914
**Aspartate aminotransferase (U/L)**			
Control group	25.93 ± 1.91	29.80 ± 5.83	0.008
Exercise group	26.00 ± 9.54	26.00 ± 6.56	1.000
Nutrition group	31.10 ± 13.49	38.50 ± 16.88	0.075
Combined group	26.60 ± 10.85	33.40 ± 8.26	0.153
**Urea (mmol/L)**			
Control group	5.15 ± 1.79	5.86 ± 1.66	0.100
Exercise group	5.91 ± 2.96	7.11 ± 1.29	0.347
Nutrition group	5.11 ± 1.40	6.09 ± 1.36	0.002
Combined group	5.39 ± 1.89	6.52 ± 2.46	0.474
**Creatinine (umol/L)**			
Control group	69.60 ± 15.31	80.54 ± 11.15	0.000
Exercise group	70.60 ± 17.19	83.17 ± 24.63	0.261
Nutrition group	73.57 ± 11.72	84.99 ± 8.83	0.001
Combined group	75.73 ± 19.44	95.97 ± 27.01	0.081
**Uric acid (umol/L)**			
Control group	283.07 ± 63.96	279.56 ± 74.46	0.764
Exercise group	348.33 ± 28.86	374.27 ± 61.35	0.635
Nutrition group	307.03 ± 55.62	304.84 ± 66.09	0.828
Combined group	310.18 ± 68.19	324.82 ± 74.43	0.589

After the intervention, the levels of Aspartate aminotransferase and creatinine in the control group increased, while the levels of urea and creatinine in the nutrition group increased, but the value before and after the intervention was within the normal range. There was no significant difference in other liver and kidney function indexes before and after intervention.

### Intervention compliance and adverse events

During the study, the subjects in the exercise group and combined group actually participated in exercise at least 4 times a week. The subjects in the nutrition group and combined group took the enteral nutritional preparation twice a day for at least 5 days a week. Subjects who completed the study had good compliance with both exercise and nutrition intervention.

During the intervention, two subjects in the nutrition group had abdominal distension after taking the preparation, which were relived within 1 day. No other adverse events occurred.

## Discussion

Studies have shown that exercise and nutritional intervention may effectively improve muscle strength and function and improve physical activity in the older population [10, 11]. The combined intervention of exercise and nutrition can significantly improve the muscle strength of the lower extremities of the older people in Asia with sarcopenia compared with the exercise intervention alone [12]. Many guidelines regard exercise and nutrition interventions as important measures to prevent and treat sarcopenia [2, 13].

Dietary and nutritional status are closely related to the occurrence of sarcopenia. A higher quality diet was associated with better physical performance among older adults [14]. The older population with sarcopenia have a lower intake of proteins than their non-sarcopenic peers [15, 16]. Increasing protein intake, especially at breakfast and lunch, contributes to alleviating muscle loss with aging [17]. It is recommended that each meal should provide 20 to 30 g protein, to achieve maximum stimulation of the muscle protein fractional synthetic rate [18]. In this study, the average protein intake of older people with sarcopenia before intervention was approximately 0.8 g/ (kg·d), which was much lower than the recommended intake.

Oral supplementation with protein-rich dietary supplements may play an important role in the prevention and control of sarcopenia [19]. The findings of a meta-analysis suggest that dairy proteins, at an amount of 14 to 40 g can significantly increase the AMM in middle-aged and older adults without a significant clinical effect on handgrip strength and leg press [20]. Oral protein supplementation or protein-rich nutritional supplements combined with exercise intervention can improve muscle mass and strength and improve activity in the older people [19, 21–25], and protein supplements for sarcopenic older adults along with exercise showed a larger effect size than exercise alone and no intervention [24]. Study showed that oral whey protein-rich supplements could significantly improve the SMM, muscle strength and function, and this effect was not related to the time of oral whey protein supplements [26]. There were also studies using amino acid preparations such as leucine and L-citrulline to intervene in older people and found that some amino acid preparations could improve walking speed and lean body mass index and improve muscle mass and physical function in the older population [27, 28].

However, some studies suggest that increasing nutrition intervention while exercise intervention does not have additional benefits for the body composition, muscle strength and function of the older population [29]. Compared to exercise or nutritional intervention alone, interventions that combined exercise and nutrition may not confer additional beneficial effects on body composition, grip strength, walking speed, physical performance, or metabolic and inflammatory biomarkers in the sarcopenic obesity population [30, 31]. The existing research results were not consistent regarding whether nutrition intervention can improve muscle mass, strength and function of older people with sarcopenia, and for subjects with good nutritional status, the effect of nutrition intervention may not be significant [32]. Additionally, the effect of nutrition intervention may also be related to the formula and dose of the intervention preparation, the daily dietary intake of the subjects, the duration of intervention and other factors.

Oligopeptides are the hydrolysates of proteins. The molecular weight of oligopeptides is smaller than that of proteins, so they are easier to digest and absorb, and have some physiological functions. Some researchers believe that oligopeptides may have a better effect on sarcopenia intervention in the older population than proteins. Some in vitro and animal experiments have shown that oligopeptides may play a role in slowing the degradation of proteins in cells, promoting protein synthesis and improving muscle atrophy in animals [33, 34]. The combination of wheat oligopeptides, soybean oligopeptides and pea oligopeptides can effectively improve the reduction in muscle protein content in C2C12 cells caused by TNF-α, reduce protein degradation and promote protein synthesis by regulating the expression of E3 ubiquitin ligase and improving the protein phosphorylation level of Akt/mTOR [34]. An animal experiment found that corn oligopeptides can increase grip strength and improve the muscle atrophy induced by dexamethasone in mice [33]. In a randomized double-blind placebo-controlled trial, a protein supplement rich in collagen peptides was used to intervene in male sarcopenia patients with resistance exercise. After 12 weeks of intervention, the fat free mass, isokinetic quadriceps strength and sensory motor control of all subjects were significantly increased, the fat mass was significantly decreased, and the effect of the intervention group treated with oral administration of 15 g collagen peptide per day was more significant [35]. Based on the results of our previous studies [33, 34], a preparation with plant oligopeptides as the main protein source was designed, which was used in this study to intervene in sarcopenia older patients alone or in combination with exercise to verify the effect of the oligopeptide preparation alone or in combination with exercise intervention in sarcopenia. In this study, oral oligopeptide nutrition preparation and exercise were used to intervene the older people with sarcopenia.

After 16 weeks of intervention, it was found that the weight and grip strength of the older people receiving exercise intervention increased, while the weight, grip strength and gait speed of the subjects receiving nutritional intervention increased. However, neither exercise nor nutrition intervention alone could maintain the fat free mass of older people. Only the subjects in the combined group maintained the muscle mass and fat free mass of each segment after the intervention.

Low-grip strength is predictive of mortality, longer hospital stays, and limited physical function. The handgrip strength is rarely used as the indicator for the detection and diagnosis of clinical diseases. However, studies have shown that grip strength is one of the important indicators for evaluating muscle strength, and it is also closely related to cognitive function, the occurrence of tumors, diabetes and frailty, the mortality of older people and the prognosis of hospitalized patients [36–41]. Low HGS was associated with a higher mortality risk in male older hospitalized patients. In medical inpatients at nutritional risk, each decrease of 10 kg in HGS was associated with increased risk of 30-d mortality and 180-d mortality [42]. Higher baseline GS and 5-year increase in GS were protective of mortality, whilst GS decline was associated with an increased risk of mortality in the very old over 9.6 years, especially in women. Men and women with a negative slope had a 16 and 33% increased risk of mortality, respectively, with every kg/year decline in GS (*P* ≤ 0.005) [43]. In this study, the right handgrip strength of the combined group increased from23.06 ± 7.05kg to 25.11 ± 6.56kg, and the left handgrip strength increased form 21.91 ± 6.21kg to 24.65 ± 6.56kg. We believe that such an increase is of practical health benefits for the subjects. Gait speed can be useful in predicting falls and expected survival, and might be included as part of a comprehensive evaluation for older adults [44–46]. In fact, many frailty or functional disability studies define slowness as 6-m walk < 1.0 m/s. Therefore, in this study, the gait speed of the nutrition group increased from 0.96 ± 0.23m/s to 1.10 ± 0.21m/s, and we believe that such an increase is of practical health benefits for the subjects.

In this study, no increase in muscle mass or SMI was observed in any intervention group, which may be related to the low daily dietary protein intake of the subjects. Some studies suggest that the effect of nutrition intervention on muscle mass is related to the nutritional status and dietary intake of the subjects, and the effect may be better in subjects with good nutritional status and adequate dietary protein intake. During the intervention, all the subjects received individualized diet guidance and nutrition education provided by professional dietitians and provided individualized recipes and recommendations for dietary intake. However, although the dietary intake of the subjects increased at the end of the intervention, the dietary protein intake did not reach the recommended amount of 1.2 g/(kg·d). Only subjects of the nutrition group and combined group, whose total average daily protein intake reached 1.34 g/(kg·d) and 1.29 g/(kg·d), respectively, reaching the recommended intake amount.

It is suggested that although dietary guidance intervention for the older people has effect on increasing dietary intake, it is difficult to change its natural dietary pattern and achieve the ideal intervention effect. A study compared the effects of high protein dietary supplements (Supp group) and adequate protein intake dietary guidance (Diet group) on maintaining muscle mass and strength in the older people with sarcopenia. The results showed that the total energy and protein intake of both groups increased after intervention but the intake of Supp group was higher than that of Diet group. The AMMI increased in the older people with sarcopenia as long as they consumed enough protein per day (1.2–1.5 g per kilogram of body weight). However, compared with dietary guidance, nutritional supplementation makes it easier for the older population with sarcopenia to achieve the sufficient protein they need [47]. The results are similar to those of this study.

The effects of exercise intervention on muscle mass, muscle strength and physical function in sarcopenia subjects may be related to different exercise types, intensity, duration, and whether to combine other intervention measures [11]. A study used resistance exercise to intervene in older women with sarcopenia for 16 weeks and found that the physical function, grip strength and walking speed of the subjects increased, but no increase in muscle mass was observed [48]. Another study used low-intensity and moderate-intensity resistance training to intervene in people aged 50 to 79 years old in the community. After 24 weeks of intervention, an increase in the cross-sectional area of contractile muscle was observed in the moderate-intensity exercise group, while only an increase in the cross-sectional area of the quadriceps femoris was observed in the low-intensity exercise group [49]. The exercise intervention duration of studies in the older people with sarcopenia usually lasted between 3 and 18 months, and improvements in muscle strength and function could be observed at the end of the study, but only a few studies could observe an increase in muscle mass [50].In this study, exercise intervention increased the muscle strength of the subjects, but the muscle mass did not increase significantly. The muscle mass of the subjects in the exercise group showed a decreasing trend. Only for the subjects in the combined group who received both exercise and nutrition interventions, the muscle mass observed to maintain preintervention levels at the end of the study. The reason may be related to the duration and intensity of intervention, the compliance of subjects, and the dietary intake and nutrition status of subjects.

There were still some limitations in this study. First, the exercise intervention was used in this study, so the subjects and researchers were difficult to be blinded. Second, there was no whey protein intervention group, so it didn’t compare the effect of oligopeptides and whey protein on sarcopenia. For sarcopenia patients without other diseases, it is not clear whether oligopeptides have advantages compared with whey protein, but for some older people with poor digestive function and intolerance to whole protein preparations, the oligopeptide nutrition preparation in this study may have important implications. Further research will be conducted on the older population with sarcopenia and poor digestive function. Third, some subjects were not willing to accept the intervention according to the plan, so there were some subjects with poor compliance and high loss rate. Fourth, the intervention period of 16 weeks may be too short to observed the increase of skeletal muscle mass in the subjects, and longer intervention should be conducted to explore the effects of exercise and nutrition intervention on skeletal muscle mass. Fifth, in this study, we used the Segmental Multi-Frequency BIA to measure the body composition of the subjects, using the Inbody 770 instrument. MRI, CT, and DXA were not used in this study in the older people due to cost, accessibility, and the problem of radiation exposure. But we found in the literature that the correlation between appendicular skeletal muscle mass obtained by using BIA and DXA was high in both men and women. The Pearson correlation coefficient and standard error of the estimate of the regression equation were 0.94 and 1.05 kg in the men, and 0.90 and 0.93 kg in the women, respectively (both *p* > 0.05). No significant method-related biases were found [51]. The concordance correlation coefficient between fat free mass measured by using DXA and BIA was 0.98(95%CI 0.97,0.98) [52]. Sixth, the results of some studies show that the increase in muscle mass, strength, and physical function of the lower extremity may be more obvious after intervention [53, 54], but the outcome indicators reflecting muscle strength and function in this study were relatively simple, so the intervention effect may be underestimated.

## Conclusions

This study used oligopeptide preparation and exercise alone or in combination to intervene in the older people with sarcopenia in the community. The results showed that exercise and nutrition intervention could improve the muscle strength or function of the older population, but short-term intervention could not alleviate the declining trend of muscle mass. Only the older people in the combined group who received both nutrition and exercise interventions maintained the muscle mass after the intervention. At the same time, for the older people with insufficient energy and protein intake, dietary guidance is not ideal to increase intake, and oral nutritional supplements should be actively adopted.

### Supplementary Information

**Supplementary Material 1. **

## Data Availability

The dataset used during this study are available from the corresponding author upon reasonable request.

## Abbreviations

MNA-SF: Mini nutritional assessment short-form
BMI: Body mass index
SLM: Soft lean mass
SMM: Skeletal muscle mass
BMR: Basal metabolic rate
ASMM: Appendicular skeletal muscle mass
SMI: Appendicular skeletal muscle mass index
FFM: Fat free mass
BCM: Body cell mass

## References

[1] Chen L-K, Liu L-K, Woo J, Assantachai P, Auyeung T-W, Bahyah KS, Chou M-Y, Chen L-Y, Hsu P-S, Krairit O (2014). Sarcopenia in Asia: Consensus Report of the Asian Working Group for Sarcopenia. J Am Med Dir Assoc.

[2] Chen L-K, Woo J, Assantachai P, Auyeung T-W, Chou M-Y, Iijima K, Jang HC, Kang L, Kim M, Kim S (2020). Asian Working Group for Sarcopenia: 2019 Consensus Update on Sarcopenia Diagnosis and Treatment. J Am Med Dir Assoc.

[3] Chen Z, Li WY, Ho M, Chau PH (2021). The Prevalence of Sarcopenia in Chinese Older Adults: Meta-Analysis and Meta-Regression. Nutrients.

[4] Yu S, Umapathysivam K, Visvanathan R (2014). Sarcopenia in older people. Int J Evid Based Healthc.

[5] Juan L, Qingqing D, Baiyu Z, Xiang L, Jingmin L, Yongming L, Guoxian D, Cuntai Z, Jianye W, Pulin Y (2022). Chinese expert consensus on diagnosis and treatment for elderly with sarcopenia(2021). J Cachexia Sarcopenia Muscle.

[6] Kang L, Gao Y, Liu X, Liang Y, Chen Y, Liang Y, Zhang L, Chen W, Pang H, Peng L-N (2019). Effects of whey protein nutritional supplement on muscle function among community-dwelling frail older people: A multicenter study in China. Arch Gerontol Geriatr.

[7] Morteza T, Irandoust K, Moddaberi S (2019). The effects of weight-bearing exercise on postural control and fatigue index of elderly males. Int Arch Health Sci.

[8] Rizia S, Cristiellem R, Lucas G, Rodrigo V, Douglas S, Claudio L, Marilia A, Beat K, Pantelis N, Meiry O (2022). Motivation for Brazilian older adult women to join a community physical activity program before COVID-19 pandemic. Int J Sport Studies Health.

[9] Kwok TC, Bai X, Li JC, Ho FK, Lee TM (2013). Effectiveness of cognitive training in Chinese older people with subjective cognitive complaints: a randomized placebo-controlled trial. Int J Geriatr Psychiatry.

[10] Yoshimura Y, Wakabayashi H, Yamada M, Kim H, Harada A, Arai H (2017). Interventions for Treating Sarcopenia: A Systematic Review and Meta-Analysis of Randomized Controlled Studies. J Am Med Dir Assoc.

[11] Negm AM, Lee J, Hamidian R, Jones CA, Khadaroo RG (2022). Management of Sarcopenia: A Network Meta-Analysis of Randomized Controlled Trials. J Am Med Dir Assoc.

[12] Li L, He Y, Jin N, Li H, Liu X (2022). Effects of protein supplementation and exercise on delaying sarcopenia in healthy older individuals in Asian and non-Asian countries: A systematic review and meta-analysis. Food Chem X.

[13] Cui H, Wang Z, Wu J, Liu Y, Zheng J, Xiao W, He P, Zhou Y, Wang J, Yu P (2023). Chinese expert consensus on prevention and intervention for elderly with sarcopenia (2023). Aging Med (Milton).

[14] Bloom I, Shand C, Cooper C, Robinson S, Baird J (2018). Diet Quality and Sarcopenia in Older Adults: A Systematic Review. Nutrients.

[15] Tessier A-J, Chevalier S (2018). An Update on Protein, Leucine, Omega-3 Fatty Acids, and Vitamin D in the Prevention and Treatment of Sarcopenia and Functional Decline. Nutrients.

[16] Coelho-Junior HJ, Calvani R, Azzolino D, Picca A, Tosato M, Landi F, Cesari M, Marzetti E (2022). Protein Intake and Sarcopenia in Older Adults: A Systematic Review and Meta-Analysis. Int J Environ Res Public Health.

[17] Smeuninx B, Greig CA, Breen L (2020). Amount, Source and Pattern of Dietary Protein Intake Across the Adult Lifespan: A Cross-Sectional Study. Front Nutr.

[18] Deutz NE, Wolfe RR (2013). Is there a maximal anabolic response to protein intake with a meal?. Clin Nutr.

[19] Bo Y, Liu C, Ji Z, Yang R, An Q, Zhang X, You J, Duan D, Sun Y, Zhu Y (2019). A high whey protein, vitamin D and E supplement preserves muscle mass, strength, and quality of life in sarcopenic older adults: A double-blind randomized controlled trial. Clin Nutr.

[20] Hanach NI, McCullough F, Avery A (2019). The impact of dairy protein intake on muscle mass, muscle strength, and physical performance in middle-aged to older adults with or without existing sarcopenia: a systematic review and meta-analysis. Adv Nutr.

[21] Kemmler W, Kohl M, Jakob F, Engelke K, von Stengel S (2020). Effects of high intensity dynamic resistance exercise and whey protein supplements on Osteosarcopenia in older men with low bone and muscle mass final results of the randomized controlled frost study. Nutrients.

[22] Liao C-D, Liao Y-H, Liou T-H, Hsieh C-Y, Kuo Y-C, Chen H-C (2021). Effects of protein-rich nutritional composition supplementation on sarcopenia indices and physical activity during resistance exercise training in older women with knee Osteoarthritis. Nutrients.

[23] Nilsson MI, Mikhail A, Lan L, Di Carlo A, Hamilton B, Barnard K, Hettinga BP, Hatcher E, Tarnopolsky MG, Nederveen JP (2020). A five-ingredient nutritional supplement and home-based resistance exercise improve lean mass and strength in free-living elderly. Nutrients.

[24] Liao C-D, Chen H-C, Huang S-W, Liou T-H (2019). The role of muscle mass gain following protein supplementation plus exercise therapy in older adults with sarcopenia and frailty risks: A systematic review and meta-regression analysis of randomized trials. Nutrients.

[25] Cruz-Jentoft AJ, Bahat G, Bauer J, Boirie Y, Bruyère O, Cederholm T, Cooper C, Landi F, Rolland Y, Sayer AA (2019). Sarcopenia: Revised European consensus on definition and diagnosis. Age Ageing.

[26] Nabuco H, Tomeleri C, Sugihara Junior P, Fernandes R, Cavalcante E, Antunes M, Ribeiro A, Teixeira D, Silva A, Sardinha L (2018). Effects of whey protein supplementation pre- or post-resistance training on muscle mass, muscular strength, and functional capacity in pre-conditioned older women: a randomized clinical trial. Nutrients.

[27] Martínez-Arnau FM, Fonfría-Vivas R, Buigues C, Castillo Y, Molina P, Hoogland AJ, van Doesburg F, Pruimboom L, Fernández-Garrido J, Cauli O (2020). Effects of Leucine administration in Sarcopenia: a randomized and placebo-controlled clinical trial. Nutrients.

[28] Caballero-García A, Pascual-Fernández J, Noriega-González DC, Bello HJ, Pons-Biescas A, Roche E, Córdova-Martínez A (2021). L-Citrulline Supplementation and Exercise in the Management of Sarcopenia. Nutrients.

[29] Choi M, Kim H, Bae J (2021). Does the combination of resistance training and a nutritional intervention have a synergic effect on muscle mass, strength, and physical function in older adults? A systematic review and meta-analysis. BMC Geriatr.

[30] Hsu KJ, Liao CD, Tsai MW, Chen CN (2019). Effects of exercise and nutritional intervention on body composition, metabolic health, and physical performance in adults with Sarcopenic obesity: a meta-analysis. Nutrients.

[31] Wu P-Y, Huang K-S, Chen K-M, Chou C-P, Tu Y-K (2021). Exercise, nutrition, and combined exercise and nutrition in older adults with Sarcopenia: a systematic review and network meta-analysis. Maturitas.

[32] Beaudart C, Dawson A, Shaw SC, Harvey NC, Kanis JA, Binkley N, Reginster JY, Chapurlat R, Chan DC, Bruyère O (2017). Nutrition and physical activity in the prevention and treatment of sarcopenia: systematic review. Osteoporos Int.

[33] Wang Y, Yan J, Zhou Z, Guo J, Wei Y, Jing X, Liao X, Hu W (2023). Effect of corn oligopeptide on dexamethasone-induced muscle atrophy. West Chin Med J.

[34] Gaiping C, Mingliang L, Jing L, Miao X, Ke L, Jiuming Y, et al. Effects of wheat oligopeptides and its combination with soybean oligopeptides and pea oligopeptides on protein expression in C2C12 skeletal muscle cells stimulated by TNF-α in vitro. Food and Fermentation Industries. 2023;50(02):153–158.

[35] Zdzieblik D, Oesser S, Baumstark MW, Gollhofer A, Konig D (2015). Collagen peptide supplementation in combination with resistance training improves body composition and increases muscle strength in elderly sarcopenic men: a randomised controlled trial. Br J Nutr.

[36] Pao YC, Chen CY, Chang CI, Chen CY, Tsai JS (2018). Self-reported exhaustion, physical activity, and grip strength predict frailty transitions in older outpatients with chronic diseases. Medicine (Baltimore).

[37] Yeung CHC, Au Yeung SL, Fong SSM, Schooling CM (2019). Lean mass, grip strength and risk of type 2 diabetes: a bi-directional Mendelian randomisation study. Diabetologia.

[38] Parra-Soto S, Pell JP, Celis-Morales C, Ho FK (2022). Absolute and relative grip strength as predictors of cancer: prospective cohort study of 445552 participants in UK Biobank. J Cachexia Sarcopenia Muscle.

[39] Moon JH, Kim YJ, Oh YH, Kong MH, Kim HJ (2019). Association between Colorectal Adenoma and Hand Grip Strength in the Elderly. J Bone Metab.

[40] Sternäng O, Reynolds CA, Finkel D, Ernsth-Bravell M, Pedersen NL, Dahl Aslan AK (2016). Grip strength and cognitive abilities: associations in old Age. J Gerontol B Psychol Sci Soc Sci.

[41] Chua KY, Lim WS, Lin X, Yuan JM, Koh WP (2020). Handgrip strength and timed Up-and-Go (TUG) test are predictors of short-term mortality among elderly in a population-based cohort in Singapore. J Nutr Health Aging.

[42] Scheerman K, Meskers CGM, Verlaan S, Maier AB (2021). Sarcopenia, Low Handgrip Strength, and Low Absolute Muscle Mass Predict Long-Term Mortality in Older Hospitalized Patients: An Observational Inception Cohort Study. J Am Med Dir Assoc.

[43] Granic A, Davies K, Jagger C, Dodds RM, Kirkwood TBL, Sayer AA (2017). Initial level and rate of change in grip strength predict all-cause mortality in very old adults. Age Ageing.

[44] Beck Jepsen D, Robinson K, Ogliari G, Montero-Odasso M, Kamkar N, Ryg J, Freiberger E, Masud T (2022). Predicting falls in older adults: an umbrella review of instruments assessing gait, balance, and functional mobility. BMC Geriatr.

[45] Marengoni A, Bandinelli S, Maietti E, Guralnik J, Zuliani G, Ferrucci L, Volpato S (2017). Combining Gait speed and recall memory to predict survival in late life: population-based study. J Am Geriatr Soc.

[46] Sanders JB, Bremmer MA, Comijs HC, van de Ven PM, Deeg DJH, Beekman ATF (2017). Gait Speed and Processing Speed as Clinical Markers for Geriatric Health Outcomes. Am J Geriatr Psychiatry.

[47] Lin CC, Shih MH, Chen CD, Yeh SL (2021). Effects of adequate dietary protein with whey protein, leucine, and vitamin D supplementation on sarcopenia in older adults: An open-label, parallel-group study. Clin Nutr.

[48] Seo M-W, Jung S-W, Kim S-W, Lee J-M, Jung HC, Song J-K (2021). Effects of 16 weeks of resistance training on muscle quality and muscle growth factors in older adult women with Sarcopenia: a randomized controlled trial. Int J Environ Res Public Health.

[49] Otsuka Y, Yamada Y, Maeda A, Izumo T, Rogi T, Shibata H, Fukuda M, Arimitsu T, Miyamoto N, Hashimoto T (2022). Effects of resistance training intensity on muscle quantity/quality in middle-aged and older people: a randomized controlled trial. J Cachexia Sarcopenia Muscle.

[50] Cruz-Jentoft AJ, Landi F, Schneider SM, Zuniga C, Arai H, Boirie Y, Chen LK, Fielding RA, Martin FC, Michel JP (2014). Prevalence of and interventions for sarcopenia in ageing adults a systematic review report of the international Sarcopenia initiative (EWGSOP and IWGS). Age Ageing.

[51] Wang H, Hai S, Cao L, Zhou J, Liu P, Dong BR (2016). Estimation of prevalence of sarcopenia by using a new bioelectrical impedance analysis in Chinese community-dwelling elderly people. BMC Geriatr.

[52] Hurt RT, Ebbert JO, Croghan I, Nanda S, Schroeder DR, Teigen LM, Velapati SR, Mundi MS (2021). The Comparison of segmental multifrequency bioelectrical impedance analysis and dual-Energy X-ray Absorptiometry for estimating fat free mass and percentage body fat in an ambulatory population. JPEN J Parenter Enteral Nutr.

[53] Lu L, Mao L, Feng Y, Ainsworth BE, Liu Y, Chen N (2021). Effects of different exercise training modes on muscle strength and physical performance in older people with sarcopenia: a systematic review and meta-analysis. BMC Geriatr.

[54] Zhu LY, Chan R, Kwok T, Cheng KC, Ha A, Woo J (2019). Effects of exercise and nutrition supplementation in community-dwelling older Chinese people with sarcopenia: a randomized controlled trial. Age Ageing.

