# Supplementary material for: Effects of oral oligopeptide preparation and exercise intervention in older people with sarcopenia: a randomized controlled trial
Source: BMC Geriatr. 2024 Mar 18;24:260. doi: 10.1186/s12877-024-04860-2 (PMC10946144; doi:10.1186/s12877-024-04860-2)
Supplement: Supplementary file 1 — Supplementary Material 1. [file 12877_2024_4860_MOESM1_ESM.docx]

Effects of oral oligopeptide preparation and exercise intervention in older people with sarcopenia: a randomized controlled trial

**Details of the trial protocol**

**Method**

**Study design**

This is a multicenter randomized controlled study. The participants of this study were older residents with sarcopenia in rural areas of Chengdu, Sichuan Province.

The community doctors assisted researchers in recruiting subjects. SPSS 27.0 was used to generate a random number sequence. Subjects who met the inclusion criteria were grouped according to the random number. A total of 219 elderly with sarcopenia were randomly divided into 4 groups: the control group was given individualized nutrition education provided by professional dietitians; the nutrition group was given individualized nutrition education and oral enteral nutrition preparations rich in oligopeptides; the exercise group received exercise intervention and individualized nutrition education; and the combined group received both oral enteral nutrition intervention and exercise intervention. The intervention lasted for 16 weeks. The researchers scheduled face-to-face interviews every four weeks as well as phone calls every week to follow up the compliance. Due to the exercise intervention used in this study, the subjects and researchers were not blinded.

A 24-hour dietary survey was conducted at the baseline and end of the intervention. Muscle strength, physical function and body composition were measured before and after the intervention, and fasting blood samples were collected before and after the intervention for hematological examination, such as routine blood tests, fasting blood glucose, blood lipids and liver and kidney function.

This study was approved by the Ethics Committee on Biomedical Research, West China Hospital of Sichuan University and registered at https://www.chictr.org.cn/ (ChiCTR2100052135). All participants provided written informed consent.

**Outcomes**

The primary outcome was the change in grip strength and 6-m walking speed before and after intervention. The secondary outcomes included the change of dietary intake, body composition and hematological indexes between the baseline and after intervention.

**Participants**

The participants of this study were 219 older residents with sarcopenia in rural areas of Chengdu, Sichuan Province. The inclusion criteria were as follows:(1) ≥ 65 years old; (2) in accordance with the diagnostic criteria of the Asian Working Group for Sarcopenia (AWGS) for sarcopenia in 2019; (3) ability to eat independently; (4) no significant weight loss (< 10%) in the past 6 months; and (5) signed informed consent form.

The exclusion criteria were as follows: (1) those who are intolerant to enteral nutrition or allergic to the composition of the tested samples; (2) patients with severe digestive and absorption diseases of the enteral preparation used in this study; (3) patients with severe heart, liver, kidney, lung and other organic diseases and/or severe dysfunction; (4) patients with lower extremity joint diseases or dysfunction affecting independent walking; (5) patients with metal or electronic devices in the body that affect the examination of body composition; (6) other nutritional agents and drugs (such as hormones, antitumour drugs, antituberculosis drugs, sedation or other muscle relaxants, etc.) that may affect the effectiveness of the test are being used; (7) those who practiced resistance training previously; (8)refusing to sign informed consent or other substances considered by the researcher to be unsuitable for this study.

The withdrawal criteria were as follows: (1) the participants who could not complete the intervention or lost to follow-up; (2) those who had serious adverse effects during the study and cannot continue to receive the intervention; (3) participants who were diagnosed with diseases that met the exclusion criteria during the study.

**Nutrition intervention and supplement protocol**

At the beginning of the study, each subject received nutrition education provided by professional dietitians. According to the basic metabolic rate, combined with the age, gender and physical activity of the subjects, the daily energy and protein requirements were determined. We calculated the energy and protein requirements by ideal body weight. The ideal weight was calculated using the following formula: ideal weight(kg) = [height(cm) - 100] × 0.9. The energy requirement was 25-30 kcal/(kg·d) and the protein requirement was 1.2-1.5 g/(kg·d). The proportion of high-quality protein was over 60%, and the protein was evenly distributed among the three meals. According to the daily requirements of energy and protein, each subject was assigned an individualized daily reference diet by professionally trained dietitians.

The enteral nutrition preparation used in this study was with plant oligopeptides as the main protein sources developed in the previous study. The preparation contained 370 kcal energy, 32g casein peptide, 10.5g pea peptide, 10g branch chain amino acid, 7.5g corn oligopeptide, and 5 g Ca-HMB per 100 g. The subjects in nutrition group and combined group received nutrition intervention, oral enteral nutrition preparation 1 pack each time (25g) for twice a day, and can provide energy 185 kcal, protein 24.2 g (including plant oligopeptide 11 g, casein peptide 4g, branch chain amino acid 5 g), CaHMB2.5 g per day. The total duration of the intervention was 16 weeks.

**Exercise intervention**

The exercise program used in this study was designed by professional rehabilitation doctors considering the physical activity ability of the elderly with sarcopenia, including 10-15min warm-up exercise, 30min resistance exercise and 20 min aerobic exercises and relaxation. The resistance exercise needs to be completed by dumbbells. The weight of the dumbbell was determined by the physical function of subjects, in a range of 2 to 10 pounds. The intensity of the resistance training was low initially to reduce muscle soreness and then progressed by increasing the amount of weight lifted until participants could complete 12 repetitions for each group.

Subjects of the exercise group and combined group received the exercise intervention. The exercise intervention was organized by community doctors and assisted by staff with experience in sports rehabilitation. Exercise was performed 5 times a week in the activity room of the community health service center, each time lasting about 60 minutes.

**Dietary survey and nutrition assessment**

The nutritional status of the elderly was evaluated by the MNA-SF before intervention by professionally trained dieticians. A 24-hour dietary survey was conducted at the beginning and end of the intervention. The dietary survey was conducted by trained dietitian. The subjects described the types and intakes of all foods consumed during the previous day. Food models and standard household measures (bowls, cups and spoons) were used to estimate portion sizes. The daily total energy, protein and fat intake were calculated.

**Anthropometric measurement**

The height and weight were measured with a corrected height and body weight meter (Wuxi weighing instrument Factory Co., Ltd., RGZ-120-RT). During the measurement, the subjects needed to fast, defecate empty, remove their shoes and hats and thick coats, and wear light to improve the accuracy of the measurement.

**Body composition**

The body composition of the subjects was analysed by bioelectrical impedance analysis (Inbody770) before and after the intervention. Data on skeletal muscle mass (SMM), appendicular skeletal muscle mass (ASMM), fat free mass (FFM), Soft lean mass (SLM) and body cell mass (BCM) were obtained. During the measurement, the subjects needed to be fasting, empty defecation, wearing light clothes, removing all the metal items and electronic products they carried, and removing shoes and socks.

**Muscular strength and function**

The grip strength and 6-m normal walking speed of the subjects were measured before and after the intervention. Grip strength was measured by the CAMRY grip meter to reflect the muscle strength of the subjects. During the measurement, the subjects were standing with full elbow extension, the grip strength meter was not in contact with the body and clothing. The grip strength was measured 3 times and the maximum reading were recorded. Physical activity ability was reflected by measuring the 6-m walking speed of the subjects. The subjects passed the 9-metre-long test region at normal gait speed. The researchers started timing at the moment subjects passed the 3-metre-long pre-walking area. The time of passing the 6-metre distance was measured. The walking speed was measured 2 times and the faster one was recorded.

**Hematological examination**

Fasting blood was collected before and after the intervention. Routine blood tests were performed by the automatic blood analyser (M-series GP). Albumin, liver and kidney function, fasting blood glucose, blood lipids and uric acid were measured by the biochemical instrument (Olympus AU400).

**Adverse events**

During the study, if any adverse events or intestinal intolerance occurred in the subjects, they should be reported to the researchers of the study in time. The researchers decided whether to suspend the intervention or withdraw from the study and gave corresponding medical treatment if necessary.

**Follow-up**

After the subjects were included in the study, they were entered different wechat groups according to their groups. The researchers followed up the compliance with the intervention through wechat. In case of adverse events, subjects can report to the researchers via wechat at any time. The researchers conducted a telephone follow-up once a week to notice the compliance and whether adverse events occurred. The face-to-face follow-up was conducted every 4 weeks.

**Data management and statistical analysis**

The data analysis was carried out by the statistician who did not know the groups of subjects. Epidata 3.1 was used for data entry, and SPSS 27.0 was used for statistical analysis of the data. The classification data were described by rate (%), the quantitative data were described by the mean (standard deviation) [$\bar{x}$(s)]. The Pearson chi-square test, Fisher’s exact test and one-way ANOVA were used to compare the differences between the groups at baseline. The Paired Samples *t*-test was used for comparison before and after intervention. All statistical tests were two-tailed, and significance was set at *p* < 0.05.
